# dipwmsearch: a Python package for searching di-PWM motifs

**DOI:** 10.1093/bioinformatics/btad141

**Published:** 2023-04-03

**Authors:** Marie Mille, Julie Ripoll, Bastien Cazaux, Eric Rivals

**Affiliations:** LIRMM, Univ Montpellier, CNRS, Montpellier, France; LIRMM, Univ Montpellier, CNRS, Montpellier, France; LIRMM, Univ Montpellier, CNRS, Montpellier, France; LIRMM, Univ Montpellier, CNRS, Montpellier, France; Institut Français de Bioinformatique, CNRS UAR 3601, Évry, France

## Abstract

**Motivation:**

Seeking probabilistic motifs in a sequence is a common task to annotate putative transcription factor binding sites or other RNA/DNA binding sites. Useful motif representations include position weight matrices (PWMs), dinucleotide PWMs (di-PWMs), and hidden Markov models (HMMs). Dinucleotide PWMs not only combine the simplicity of PWMs—a matrix form and a cumulative scoring function—but also incorporate dependency between adjacent positions in the motif (unlike PWMs which disregard any dependency). For instance to represent binding sites, the HOCOMOCO database provides di-PWM motifs derived from experimental data. Currently, two programs, SPRy-SARUS and MOODS, can search for occurrences of di-PWMs in sequences.

**Results:**

We propose a Python package called *dipwmsearch*, which provides an original and efficient algorithm for this task (it first enumerates matching words for the di-PWM, and then searches these all at once in the sequence, even if the latter contains IUPAC codes). The user benefits from an easy installation via *Pypi* or *conda*, a comprehensive documentation, and executable scripts that facilitate the use of di-PWMs.

**Availability and implementation:**

*dipwmsearch* is available at https://pypi.org/project/dipwmsearch/ and https://gite.lirmm.fr/rivals/dipwmsearch/ under Cecill license.

## 1 Introduction

**Figure 1:**
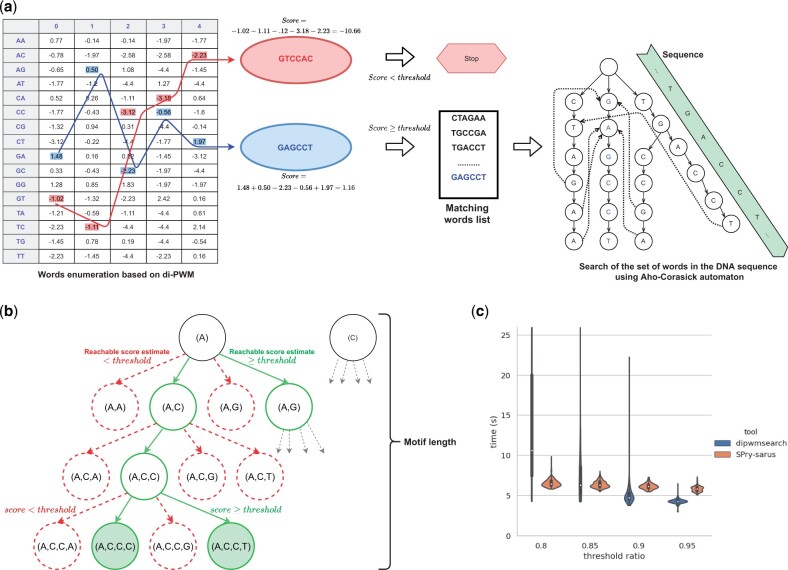
(a) Enumeration and scanning strategy for a di-PWM. Left part shows how the score of two words are computed by summing the score of their five dinucleotides. If the score lies above the threshold, the word is a valid word and is added to the list for later search. Right part: we build an Aho–Corasick automaton with all valid words in the list, then use the automaton to scan the sequence. (b) Illustration of the branch and bound strategy for the enumeration procedure. We build a trie for words starting with letter *A*, and explore it in Depth-First manner. As soon as a prefix cannot give rise to a valid word, which is determined using the LookAheadMatrix (LAM), we cut the corresponding branch. Only valid words generate a leaf in the trie. (c) Comparison of SPRy-SARUS and dipwmsearch for searching all Human di-PWMs from HOCOMOCO on Human chromosome 15. The violin plot shows the running times over for all di-PWMs and their median for both tools

Protein binding sites on nucleic acids (DNA or RNA) share similar, but not identical sequences. The collection of sequences of such binding sites, which in practice is a set of sequences (of identical length), are summarized and represented as a probabilistic motif. Often only a few positions within such sequences are conserved across a majority of their binding sites. Even at a conserved position, when the collection is large enough, alternative nucleotides occur. Hence, for each position of the binding site, it is convenient to summarize its variability as the probability of each nucleotide to occur at this position. The probabilities are estimated from the frequencies of nucleotides at that position in the collection. This explains why the first and most popular probabilistic motif representation is the Position Weight Matrix (PWM) (Stormo 2000). A PWM is a matrix containing the weight or score of each nucleotide at each position of the sequence alignment: the weights are log-odd scores of the nucleotide probabilities at each position. Numerous search algorithms are available for PWMs (Beckstette et al. 2006; Korhonen et al. 2009; Martin et al. 2018). However, in a PWM positions are entirely independent one of another; but in reality, neighboring positions are constrained since they influence the shape of DNA, or the propensity to undergo epigenetic modifications, and hence the binding of the protein. Hence, a more complex representation for probabilistic motifs that accounts for local position dependencies was proposed: the dinucleotide PWM (di-PWM) (Kulakovskiy et al. 2013). At each position, one records the frequency of all 16 possible dinucleotides (instead of four nucleotides in a PWM). A di-PWM and score computation of a word is illustrated in Fig. 1a.

For instance, the *HOCOMOCO* database (v11) stores di-PWMs of binding motifs of Human and Mouse transcription factors (292 and 257, respectively), which were directly computed from experimental ChIP-Seq data (Kulakovskiy et al. 2018). For detecting new binding sites, it was shown that di-PWMs provide enhance sensitivity compared to classical PWMs (Kulakovskiy et al. 2013). di-PWMs also obtained successful results in the DREAM-ENCODE challenge from 2017.

To our knowledge, only *SPRy-SARUS* and *MOODS* can find occurrences of di-PWMs in long sequences. *SPRy-SARUS* is an efficient standalone Java program to search for di-PWMs, which is coupled with *MoLoTool*, a web tool allowing visual inspection of occurrences in short sequences (Kulakovskiy et al. 2018). MOODS, a tool to search for PSSM/PWM, can also handle di-PWMs (Korhonen et al. 2017) (v3 available as C++ code and Python package). Both adopt a window scanning strategy, while *dipwmsearch* uses an enumeration strategy.

Hence, we provide a Python package for searching di-PWM in sequences: it can be easily installed via *conda* and offers several functions that can be used in Python programs. The user can control the minimum score of occurrences found by entering either a score threshold, a ratio threshold, or a *P*-value threshold. After the search, the user can also compute and add the *P*-value of each reported occurrence in the output file. We designed a novel search algorithm that differs from previous approaches. Running time comparisons demonstrate that our algorithm is on par with SPRy-SARUS in practice.

## 2 Search algorithm

Our package provides distinct search algorithms: an optimized scanning algorithm (OS), an enumeration-based algorithm for full di-PWMs (FE), and the core enumeration-based algorithm (CE). We described the FE and CE algorithms below, and explain OS in Supplementary Material. The CE algorithm is the most time efficient of all and, by design, uses less memory than FE.

From an algorithmic viewpoint, we aim at proposing new approaches capable of finding occurrences in long sequences (written over an alphabet containing *σ* symbols) with a limited amount of memory. Before explaining the algorithm, we give some rationale for our approach. Let us consider that in a text *T*, we seek a di-PWM *P* of size $\sigma^{2}\times (m-1)$ (for a motif of length *m*) and with a score threshold *t*. An entry P[αβ,i] gives the score of dinucleotide αβ at position *i* in the motif. For the CE algorithm, we need to restrict the matrix *P* to a subset of columns (i.e. to an interval of positions) for any interval [i..j] with 1≤i<j<m.

### 2.1 Traditional scanning algorithm and enumeration strategy

In a traditional scanning algorithm, one considers each possible window of length *m* in *T* and computes its score according to *P* (see Fig. 1a). It takes O(m×|T|) time, which is quadratic. The scanning approach implies redundant computation (for instance when processing identical or similar substrings whose score are too low) and often is inefficient. A classical speedup trick uses the LookAheadTable to stop the score computation after viewing only a prefix of the current window (Beckstette et al. 2006); it does not improve the worst-case complexity.

An alternative is to first enumerate all words of length *m* that match *P* with a score >t, which we call *valid words*, and then to search the valid words in *T* using an Aho–Corasick (AC) automaton (Aho and Corasick 1975) (or any other algorithm that solves the Set Pattern Matching problem). We implemented this in the enumeration-based algorithm for full di-PWM (FE). We call this global idea the *enumeration strategy*; it concentrates the complexity in the enumeration phase and makes the scanning efficient because it seeks only exact matches of the valid words—the scanning phase does not compute any score. Nicely, building the Aho–Corasick automaton takes linear time in the cumulated length of valid words, and scanning *T* with it takes linear time in |T|.

### 2.2 Efficiency conditions for the enumeration strategy

To be efficient, the enumeration strategy needs 1/ a fast enumeration algorithm, 2/ a set of valid words that is small enough for the AC automaton to fit in memory (i.e. to remain fast to build). Below, we exhibit an enumeration algorithm that takes linear time in the output size, and satisfies the first condition. However, the number of valid words depends on the selectivity of the di-PWM *P* with threshold *t*. The least selective position is when the scores of all 16 dinucleotides are equal. It turns out that some di-PWMs from HOCOMOCO contain positions that are not selective, i.e. in which the scores of dinucleotides are almost equally distributed. A closer examination shows in such di-PWMs nonselective positions often occur in intervals of successive positions (see Supplementary Material). For such an interval of say *f* positions, if we consider P′ the restriction of *P* to this interval, almost any possible word of length *f *+* *1 is a valid word for the di-PWM P′. This may lead to an explosion of valid words for the full di-PWM *P*.

We propose to identify selective and nonselective positions by considering the standard deviation of their scores: a large deviation means a selective position. To avoid cases with huge set of valid words, we propose to restrict *P* to an interval of selective positions, which we term the *core*. We proceed as follows: first, we compute the standard deviation of scores for all positions, then we select, by exhaustive search among all possible intervals of length at least 10, the interval with the largest average standard deviation. This interval determines the core (which is a smaller di-PWM).

### 2.3 Enumeration of valid words: B&B approach and LAM

For a full di-PWM, we propose an algorithm that explores a trie data structure of valid words using a Branch-and-Bound approach (see Fig. 1b). We build a trie that spells out prefixes of potential valid words, one letter at a time. After each letter, assume the current prefix has length *k*, we compute the partial score for this prefix. Then, we check the score for the best possible suffix of length *m–k* in an additional matrix called the LAM. If the sum of prefix and suffix scores does not reach the threshold *t*, then extensions of the current branch of the trie are unnecessary. The LookAheadMatrix (or LAM for short) is a precomputed σ×(m−1) matrix that depends only on *P*. For a position *i* in *P* and a symbol *α*, the LAM[α,i] stores the best score for a suffix starting with symbol *α* at position *i*. Supplementary Algorithm S1 computes the LAM in $O(\sigma^{2}\times (m-1))$ time. The LAM has a crucial property: for any stored score value in the LAM, there exists a word that realizes this score. This ensures that only branches of the trie corresponding to valid words are fully built by the enumeration algorithm. Moreover, the amount of computation spent between two successive valid words is bounded by 2*m*, which implies that our algorithm takes linear time in the output size.

Note that a pendant matrix to the LAM can be built symmetrically to compute the best scores of prefixes of *P*. We call this matrix, the LookBackMatrix or LBM.

After enumeration, in the search step, the set of valid words for *P* are searched for in *T* using an Aho–Corasick automaton (Aho and Corasick, 1975).

### 2.4 Adapting the enumeration strategy and search to the core

The enumeration algorithm and search phase must be adapted to use the core instead of *P*. Assume the core, denoted by *Q*, starts at position *k *+* *1 in the motif and has length *h–*1. We must enumerate words of length *h* for *Q* that are substrings of valid words of length *m* for *P*. We run the branch & bound algorithm described above to spell out words of length *h* according to *Q*, but we cannot select them on their own score (which is a sum only over *h–*1 positions!). We must use the score of a prefix of *P*, not a prefix of *Q*. Assume the current prefix *w* starts with letter *α* at position *k *+* *1 and ends with letter *β*; as score, we use score(xwy), where *x* is a highest scoring valid prefix for *P* of length *k* ending with letter *α*, and *y* a highest scoring suffix of length m−k−|w|+1 starting with letter *β*, and such that |xwy|=m. The idea behind is that *xwy* is the best possible word of length *m* with substring *w* (at positions [k+1,k+|w|+1]). The constraints on the letters are implied by the fact that successive positions of a di-PWM score overlapping dinucleotides. We use the LAM to get the contribution of *y* to this score (without knowing *y*) and we use the LBM to get that of *x* (without knowing *x*). The algorithm outputs the set of all words *w* of length *h* that occur in at least one valid word for *P* (at position [k+1,k+h+1]).

The search phase builds an AC automaton with this set and scan *T* with it. Each time a match is found, say at position *i*, it computes the score of the window of *T* between positions (i−k+1) and (i−k+m). If the scores reaches the threshold *t* it reports a match of *P* at position (i−k+1) in *T*, and its score.

## 3 Results

We compared the core enumeration algorithm to SPRy-SARUS in terms of efficiency by searching each Human di-PWM from HOCOMOCO on Human chromosomes 3 and 15 with four different score ratio thresholds (0.8, 0.85, 0.90, and 0.95). The score ratio threshold is a way to set a relative score threshold that is comparable between different di-PWMs (it was developed for PWMs—see the FAQ of JASPAR database). The score threshold *θ* is computed as follows: $\theta =({\text{score}}_{\text{max}}-{\text{score}}_{\text{min}})*{\text{ratio}+\text{score}}_{\text{min}}$, where *score*_*min*_ and *score*_*max*_ are respectively the minimum and maximum achievable scores for the input di-PWM. Note that *dipwmsearch* provides an alternative to the ratio threshold: the user can give a *P*-value threshold to limit the score of occurrences found.

First, the results shown in Fig. 1c confirm that CE algorithm is able to search for any di-PWM with reasonable score ratios. Figure 1c displays the median search time over all di-PWMs for both tools, and shows first, that *dipwmsearch* offers affordable runtimes whatever the ratio, and second that, while *dipwmsearch* takes longer times than SPRy-SARUS for ratio 0.80, it is as efficient or faster for larger ratios.

On the violin plot, the set of points for dipwmsearch is needle-like due to the variability of motif length and of information content among HOCOMOCO di-PWMs. Information poor di-PWMs generate longer list of valid words, whose enumeration and storage requires longer time and larger memory, than for information rich di-PWMs. An extreme example of information poor di-PWMs, that of GATA2, is shown in Supplementary Material; others, such as MAX_HUMAN (length 23 nuc.), can be spotted looking at the LOGO representations of di-PWMs on HOCOMOCO webpage. Below in the discussion, we emphasize that enumeration of valid words and the core-based strategy can help questioning the information content and the width of some di-PWMs.

## 4 Conclusion

Our Python package, *dipwmsearch*, provides an easy and efficient procedure to find occurrences of di-PWMs in nucleotidic sequences, and well documented snippets. It offers practical advantages compared to an existing solution (like processing IUPAC codes, or an adaptable output—see Supplementary Material). Furthermore, it can be enhanced by combining it with other Python packages (e.g. for processing compressed sequence files). Most of all, the installation is straightforward using *pypi* or *conda*. In addition, we presented an original enumeration-based search algorithm that handles di-PWMs even if they contain nonselective positions. Coping with nonselective positions was necessary to make search effective for some di-PWMs, which questions their information content, and in turn their construction process. Examining the core block determined by our CE algorithm, or comparing set of valid words with the occurrences found in practice, can help determining whether the information content and the length of a di-PWM are well adapted or could be improved.

Several perspectives come to mind. First, once enumerated, the set of valid words can be stored in a file and reused for other searches. Second, the search phase can be streamlined by using a precomputed index of the searched sequence to find valid words, which would be appropriate for a web application that needs to answer numerous di-PWM searches.

## Supplementary Material

btad141_Supplementary_Data

## References

[btad141-B1] Aho A , Corasick M. Efficient string matching: an aid to bibliographic search. Commun ACM 1975;18:333–40.

[btad141-B2] Beckstette M , Homann R, Giegerich R et al Fast index based algorithms and software for matching position specific scoring matrices. BMC Bioinformatics 2006;7:389. 10.1186/1471-2105-7-389. 16930469 PMC1635428

[btad141-B3] Korhonen J , Martinmäki P, Pizzi C et al MOODS: fast search for position weight matrix matches in DNA sequences. Bioinformatics 2009;25:3181–2. 19773334 10.1093/bioinformatics/btp554 PMC2778336

[btad141-B4] Korhonen JH , Palin K, Taipale J et al Fast motif matching revisited: high-order PWMs, SNPs and indels. Bioinformatics 2017;33:514–21. 28011774 10.1093/bioinformatics/btw683

[btad141-B5] Kulakovskiy I , Levitsky V, Oshchepkov D et al From binding motifs in chip-seq data to improved models of transcription factor binding sites. J Bioinform Comput Biol 2013;11:1340004. 23427986 10.1142/S0219720013400040

[btad141-B6] Kulakovskiy IV , Vorontsov IE, Yevshin IS et al HOCOMOCO: towards a complete collection of transcription factor binding models for human and mouse via large-scale ChIP-Seq analysis. Nucleic Acids Res 2018;46:D252–9. 29140464 10.1093/nar/gkx1106 PMC5753240

[btad141-B7] Martin D , Maillol V, Rivals E. Fast and accurate genome-scale identification of DNA-binding sites. In: *2018 IEEE International Conference on Bioinformatics and Biomedicine (BIBM)*, *Madrid, Spain*, pp. 201–5. 2018.

[btad141-B8] Stormo GD. DNA binding sites: representation and discovery. Bioinformatics 2000;16:16–23. 10812473 10.1093/bioinformatics/16.1.16

