# Supplementary material for: dipwmsearch: a Python package for searching di-PWM motifs
Source: Bioinformatics. 2023 Apr 3;39(4):btad141. doi: 10.1093/bioinformatics/btad141 (PMC10081870; doi:10.1093/bioinformatics/btad141)
Supplement: btad141_Supplementary_Data [file btad141_supplementary_data.pdf]

dipwmsearch: a python package for searching di-PWM motifs – Supplementary Material

Marie Mille, Julie Ripoll, Bastien Cazaux, Eric Rivals

February 23, 2023

Affiliations: Laboratory of Informatics, Robotics and Microelectronics (LIRMM), Université Montpellier, CNRS, Montpellier, France, Contact author: E. Rivals, *Email*: `rivals@lirmm.fr`.

Abstract
Motivation Seeking probabilistic motifs in a sequence is a common task to annotate putative transcription factor binding sites (TFBS) or other RNA/DNA binding sites. Useful motif representations include Position Weight Matrices (PWMs), dinucleotide PWMs (di-PWMs), and Hidden Markov Models (HMMs). Dinucleotide PWMs combine the simplicity of PWMs – a matrix form and a cumulative scoring function –, but also incoporate dependency between adjacent positions in the motif (unlike PWMs which disregard any dependency). For instance, to represent binding sites, the HOCOMOCO database provides di-PWM motifs derived from experimental data. Currently, two programs, SPRy-SARUS and MOODS, can search for di-PWMs in sequences.
Results We propose a Python package, *dipwmsearch*, which provides an original and efficient algorithm for this task (it first enumerates matching words for the di-PWM, and then search them at once in the sequence even if it contains IUPAC codes). The user benefits from an easy installation via *Pypi* or *conda*, a comprehensive documentation, and executable scripts that facilitate the use of di-PWMs.
Availability and Implementation: *dipwmsearch* is available at https://pypi.org/project/dipwmsearch/ and https://gite.lirmm.fr/rivals/dipwmsearch/ under Cecill license. **Supplementary information**: Supplementary data are available at *Bioinformatics* online.

1 Access to the packages, the source code and the documentation.

1. Python package: `https://pypi.org/project/dipwmsearch/`
2. Documentation: `https://rivals.lirmm.net/dipwmsearch/`
3. Conda package: `https://anaconda.org/atgc-montpellier/dipwmsearch`
4. Source code: `https://gite.lirmm.fr/rivals/dipwmsearch`
5. Contact: dipwm@lirmm.fr

2 Practical comparison of SPRy-SARUS, MOODS, and dipwmsearch

We summarize some features between the tools SPRy-SARUS (url), MOODS [3], and dipwmsearch [5], in the table below.

**Feature**	SPRy-SARUS	MOODS v3	dipwmsearch
**conda installation**	n	y	y
**pypi installation**	n	y	y
**IUPAC code (except N)**	blocking	y	y
**API programming library**	n	y	y
**output matching word**	n	y	y
**compressed sequence file**	n	y	y (via gzip/bzip python packages)
**multi FASTA**	y	y	y (example)

3 Simplicity of use: an example of python code

First, as explained in the documentation, there exist two ways of using *dipwmsearch*. First, the user can directly call the module as a script using a command line of the form:

```
python -m dipwmsearch <dipwmfile> <fastafile> <ratio>
```

Example

```
python -m dipwmsearch ANDR_HUMAN.H11DI.0.A.dpwm chr15.fa 0.935
```

Second, the user can program his/her own script using the programming interface offered by the package. To illustrate how easily one can use dipwmsearch in python, we provide an example of code for searching one di-PWM motif in a FASTA sequence for a given threshold, exactly as SPRy-SARUS would perform it. This script takes three arguments on the command line: first, the file containing the di-PWM, second, the file containing the FASTA formatted sequence, and third, the threshold ratio as a real value.

```
import sys, os
import dipwmsearch as ds
from Bio import SeqIO
from Bio.Seq import Seq

pathSeq = sys.argv[1]
pathDiPwm = sys.argv[2]
threshold = float(sys.argv[3])


# read diPWM, sequence
diP = ds.create_diPwm(pathDiPwm)
file = open(pathSeq)
seqRecord = SeqIO.read(file, "fasta")

mySeq = str(seqRecord.seq.upper())
seq = mySeq.translate(mySeq.maketrans("NMRWSYKVHDB","GGGGGGGGGGG"))

# print(threshold)

# Block optimized search
number_matches_block_opt = 0
# 1st loop to search valid words on Watson strand
for i, word, score in ds.search_block_optimized(diP, seq, threshold):
    number_matches_block_opt += 1

# reverse complement sequence
seq_rev = seq.translate(seq.maketrans("ACGT","TGCA"))
seq_rev = seq_rev[::-1]

# 2nd loop to search valid words on Crick strand
for i, word, score in ds.search_block_optimized(diP, seq_rev, threshold):
    number_matches_block_opt += 1

dipwm_name = os.path.split(pathDiPwm)[1].split(".")[0]
print(f'{dipwm_name}\t{threshold}\t{number_matches_block_opt:,}')
```

4 The LookAheadMatrix (LAM): definition and algorithm

The LAM is an additional matrix that contains look ahead scores. It is a generalisation of the LookAheadTable (proposed in [2]). We give a formal definition of the LAM below. The LAM is used in the Optimized Scanning algorithm, and in the enumeration step of both the enumeration based algorithm (FE) and the Core Enumeration algorithm (CE).

In this manuscript, we consider DNA/RNA sequences and assume the classical alphabet, denoted by Σ, is $\{A,C,G,T\}$ or $\{A,C,G,U\}$. Sequences can also include symbols from the IUPAC DNA alphabet. The symbol σ denotes the alphabet size (e.g., in general $\sigma = 4$ in the case of DNA/RNA sequences). The algorithm is valid for any fixed alphabet Σ of size σ.

For DNA motif, the di-PWM matrices consider only the classical $\{A,C,G,T\}$ alphabet, and hence in the following algorithm, we have $\sigma = 4$ (In other words, di-PWM matrices do not use non-ACGT symbols).

Definition 4.1 (*LookAheadMatrix* for di-PWM) *Let Σ be an alphabet of size σ, and let P be a di-PWM for a motif of length m. The* LookAheadMatrix *M of P is a matrix of size $\sigma \times (m-1)$, where for any i such that $0 \le i \le m-2$ and for any $d \in \Sigma$:*

$$M[d,i] := \begin{cases} \max\limits_{b\in\Sigma} P[db,i], & \text{if } i = m-2 \\ \max\limits_{b\in\Sigma}(P[db,i] + M[b,i+1]), & \text{if } 0 \le i < m-2 \end{cases}$$

Algorithm 1: MakeLookAheadMatrix: compute the LookAheadMatrix of a di-PWM P.

Input: Alphabet Σ of size σ,di-PWM matrix P of size $\sigma^2 \times (m-1)$, for a motif of length m
Output: LookAheadMatrix M of size $\sigma \times (m-1)$

$M \leftarrow$ initialized with $-\infty$;
for $d \in \{0,\dots,\sigma-1\}$ **do**
    $max \leftarrow -\infty$;
    for $b \in \{0,\dots,\sigma-1\}$ **do**
        $score \leftarrow P[db,m-2]$;
        if $score > max$ **then** $max \leftarrow score$
    $M[d,m-2] \leftarrow max$;
for $i \in \{m-3,\dots,0\}$ **do**
    for $d \in \{0,\dots,\sigma-1\}$ **do**
        $max \leftarrow -\infty$;
        for $b \in \{0,\dots,\sigma-1\}$ **do**
            $score \leftarrow P[db,i] + M[b,i+1]$;
            if $score > max$ **then** $max \leftarrow score$
        $M[d,i] \leftarrow max$
return M;

---

5 The optimized scanning algorithm (OS) and the enumeration based algorithm for full di-PWM (FE).

Both algorithms take as input a di-PWM, a sequence in which motif occurrences are sought, and a score threshold.

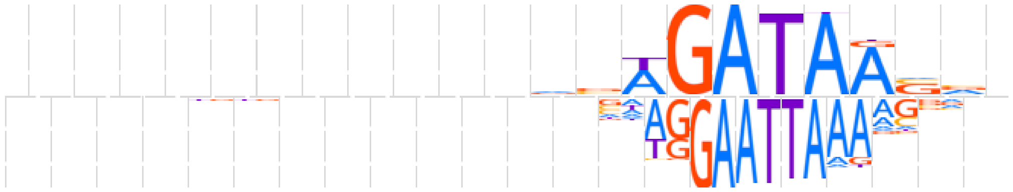

Figure 1: LOGO representation of the Human di-PWM for the binding site motif of transcription factor GATA2. The motif 23 bp long and its consensus sequence is *nnnnnnnnnnnnvvWGATAASvn*. The interval with the first 12 positions at 5' end contains non selective positions (with little information content), as is the last 3' end position. Hence, the core is restriction to interval [13, 22] of positions.

The optimized scanning algorithm (OS)

The optimized scanning algorithm (OS) follows a window scanning strategy: it scans the sequence from left to right and consider each possible window of length m. For each window, a naive algorithm would compute the score according to the di-PWM by summing the score of the current dinucleotide for each position in the window but the last. The OS algorithm iteratively computes the score of each prefix (from length 2 to m) of the window and check whether the maximal reachable score over the entire window is larger than the score threshold. If not, it stops computing scores for the current window, since the score of the current window cannot achieve the required threshold, and it continues with the next window. The maximal reachable score for a prefix of length j is obtained by summing the current prefix score with the LAM entry indexed by the last symbol of the prefix and $j+1$.

The enumeration based algorithm for full di-PWM (FE).

This strategy is radically different from the window scanning one. We first enumerate all words of length m whose score is larger than the threshold t, which we call *valid words*, and then to search the set of valid words in T using an Aho-Corasick (AC) automaton [1]. Since there is an available and efficient implementation of the Aho-Corasick (AC) automaton in Python, the key lies in the enumeration algorithm, which is described in the section entitled "Enumeration of valid words: B&B approach and LAM" of the manuscript. The properties of the LAM induces the optimality of the Branch and Bound approach when exploring the trie of words in depth first search order: indeed, the score of full words is only entirely computed for valid words. This means that the algorithm stops computing the score of a prefix of a word (exploring the corresponding branch of trie) as soon one additional symbol makes the threshold score unreachable.
The enumeration step outputs the list of valid words. A drawback is that the list can exhaust the available RAM on the computer. This occurs when the input di-PWM contains non selective columns. To avoid such a scenario, we designed the Core Optimized algorithm, which is explained in the main manuscript.

6 Non selective positions and core

An example of di-PWM with non selective positions, for GATA2 transcription factor, is shown Figure 1. The height of the letters at each motif position in the LOGO above the line, indicate the information content of the corresponding nucleotide at this position. The letters first 12 positions are nearly invisible compared to those between position 13 until 22.

7 Protocol for comparing running times

Currently, it is not possible to search for di-PWM using MOODS v3: MOODS cannot read the format for di-PWM matrices containing scores (an issue was sent on the github repository on Sep 22, 2022). This is why MOODS was not used in the comparisons.
Comparisons between dipwmsearch and SPRy-SARUS were performed as follows, using two bash scripts on a Linux system. Technical information are summarised below.

For both chromosome sequences (15 and 3), for all Human di-PWM motifs of HOCOMOCO database [4], for four ratios (0.8, 0.85, 0.9, 0.95), we recorded the running time of dipwmsearch and SPRy-SARUS using the `time` command. Each search for a di-PWM was performed in a distinct execution of the tools, implying that each time the target sequence and the di-PWM were loaded, the enumeration of valid words and then the scanning of the sequence performed, before storing the results in an output file on the disk. We did not take advantage of the possibility for dip to search for a multi FASTA file containing both sequences at once (which would have avoided to redo the enumeration step).
For dipwmsearch, we used a python script that calls the procedure `search_block_optimized` for searching the di-PWM first on the Watson strand and then on the Crick strand. The time was measured using the `time` command from Python package `time`. For SPRy-SARUS, we recorded the time taken by program in user mode, using the `/usr/bin/time` command with option `-f "%U"`. Both tools were run using a single thread; despite this, it occurs that the JAVA machine running SPRy-SARUS used more than 100% of the CPU.

Technical information

The last version of SPRy-SARUS (release: 2.0.2) was obtained from its github repository https://github.com/autosome-ru/sarus.
Linux version
Linux 5.11.0-40-generic #44~20.04.2-Ubuntu SMP Tue Oct 26 18:07:44 UTC 2021 $x86_{64}$ $x86_{64}$ $x86_{64}$ GNU/Linux
Java version

- openjdk version "10.0.2" 2018-07-17
- OpenJDK Runtime Environment Zulu10.3+5 (build 10.0.2+13)
- OpenJDK 64-Bit Server VM Zulu10.3+5 (build 10.0.2+13, mixed mode)

Variation of running time with respect to the HOCOMOCO di-PWM

The summary of results reported in the article present the running times in seconds for all HOCOMOCO di-PWM motifs. As mentioned above, the di-PWMs of HOCOMOCO vary in information content and thus in selectivity. This implies that the number of valid words strongly depends on the motif and the ratio, and hence, so do the enumeration and overall running times.
The Figures 2 provides a view on the variability of the running times in function of the di-PWM and ratio, for both SPRy-SARUS (left plot) and dipwmsearch (right plot). We report the running times for searching on Human chromosomes 15 and 3. Note that because SPRy-SARUS can only process sequences containing A, C, G, T (but no other IUPAC codes), the sequence of chromosome 3 was slightly modified to replace undetermined positions by one standard nucleotide (before feeding SPRy-SARUS). For dipwmsearch, we used the normal chromosome 3 sequence with IUPAC codes.
The variation of running times in comparison to the median running time is higher for dipwmsearch than for SPRy-SARUS. For dipwmsearch, it depends on the matrix and ratio, while SPRy-SARUS times depend mostly on the sequence length. However, the overal running times of dipwmsearch remain fast enough for practical uses.

References

[1] A. Aho and M. Corasick. Efficient string matching: an aid to bibliographic search. *Communications of the ACM*, 18:333–340, 1975.

[2] M. Beckstette, R. Homann, R. Giegerich, and S. Kurtz. Fast index based algorithms and software for matching position specific scoring matrices. *BMC Bioinformatics*, 7(1), Aug 2006.

[3] J. H. Korhonen, K. Palin, J. Taipale, and E. Ukkonen. Fast motif matching revisited: high-order PWMs, SNPs and indels. *Bioinformatics*, 33(4):514–521, Dec 2017.

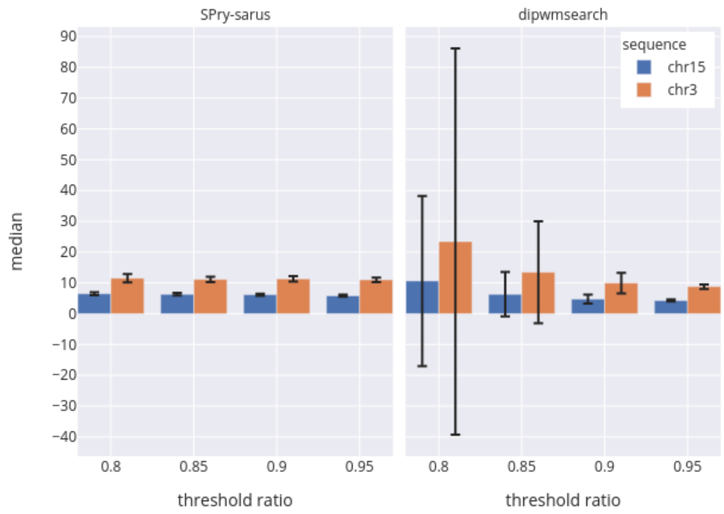

Figure 2: Median running times in seconds and standard deviations of both dipwmsearch and SPRy-SARUS for searching each Human di-PWMs from HOCOMOCO on Human chromosomes 15 and 3.

[4] I. V. Kulakovskiy, I. E. Vorontsov, I. S. Yevshin, R. N. Sharipov, A. D. Fedorova, E. I. Rumynskiy, Y. A. Medvedeva, A. Magana-Mora, V. B. Bajic, D. A. Papatsenko, and et al. HOCOMOCO: towards a complete collection of transcription factor binding models for human and mouse via large-scale ChIP-Seq analysis. *Nucleic Acids Research*, 46(D1):D252–D259, Nov 2018.

[5] M. Mille, J. Ripoll, B. Cazaux, and E. Rivals. dipwmsearch: a python package for searching di-PWM motifs. Technical report, LIRMM, Univ. Montpellier, CNRS, June 2022. HAL:lirmm-03834008.
